# The Hospital Egg: An Egg-Farmer's View

**Published:** 1915-07-17

**Authors:** 


					The Hospital Egg : An Egg-farmer's Vie*'
To the Editor of The Hospital. ^
Sir,?I notice an interesting suggestion by a , ^
spondent of The Hospital that "hospitals sho.
advantage of the present summer prices [such as
and pickle their eggs, lest worse prices should P
during the winter." The waterglass recipe that i
is an admirable one, and is the one that is 111 ff?
this egg-farm. Nor is there any doubt that ^ ^
expressed as to higher prices will be realised this ^
winter. Last winter eggs reached 3s. a dozen
districts; this year, already they are selling in. ^
mouth at 2s. a dozen, and the 4d. winter egg 15 ^
of in our local markets. How can it be
Corn, meat, and meal all show a 70 per cent-
pre-war prices, and a fowl, being by nature 0&11 ^
will not lay eggs, at any rate in winter, unlesS
sustained by a full and complete diet. gVj
And yet no housekeeper can afford to disPe
or even reduce, the egg supply?least of all 1
tional or hospital housekeeper. Quite apart
food value of an egg, and its use as a concent
easily digested nutrient, two most important
are disclosed by analysis?substances that are ^vl
scx-ibed in medicinal form in the ever-grovvi11^
nervous disorders?viz., lecithin and cerebrin. j s'j
The solution of the problem is undoubtedly
gested by The Hospital?namely, to buy i'1 1 1
April (the months that hens select above all
[Continued on p. iv.)

				

